# Monocytes and T cells incorporated in full skin equivalents to study innate or adaptive immune reactions after burn injury

**DOI:** 10.3389/fimmu.2023.1264716

**Published:** 2023-10-13

**Authors:** Patrick P.G. Mulder, Marcel Vlig, Anouk Elgersma, Lotte Rozemeijer, Leonore S. Mastenbroek, Esther Middelkoop, Irma Joosten, Hans J.P.M. Koenen, Bouke K.H.L. Boekema

**Affiliations:** ^1^ Preclinical Research, Association of Dutch Burn Centres (ADBC), Beverwijk, Netherlands; ^2^ Laboratory of Medical Immunology, Department of Laboratory Medicine, Radboud University Medical Center, Nijmegen, Netherlands; ^3^ Department of Plastic, Reconstructive and Hand Surgery, Amsterdam UMC, VU University Amsterdam, Amsterdam, Netherlands; ^4^ Tissue Function and Regeneration, Amsterdam Movement Sciences, Amsterdam, Netherlands

**Keywords:** immune response, cytokines, flow cytometry, immunohistochemistry, macrophages

## Abstract

**Introduction:**

Thermal injury often leads to prolonged and excessive inflammation, which hinders the recovery of patients. There is a notable absence of suitable animal-free models for investigating the inflammatory processes following burn injuries, thereby impeding the development of more effective therapies to improve burn wound healing in patients.

**Methods:**

In this study, we established a human full skin equivalent (FSE) burn wound model and incorporated human peripheral blood-derived monocytes and T cells.

**Results:**

Upon infiltration into the FSEs, the monocytes differentiated into macrophages within a span of 7 days. Burn-injured FSEs exhibited macrophages with increased expression of HLA-DR^+^ and elevated production of IL-8 (CXCL8), in comparison to uninjured FSEs. Among the T cells that actively migrated into the FSEs, the majority were CD4^+^ and CD25^+^. These T cells demonstrated augmented expression of markers associated with regulatory T cell, Th1, or Th17 activity, which coincided with significant heightened cytokine production, including IFN-γ, IL-4, IL-6, IL-8, IL-10, IL-12p70, IL-17A, IP-10 (CXCL10), and TGF-β1. Burn injury did not impact the studied effector T cell subsets or cytokine levels.

**Discussion:**

Collectively, this study represents a significant advancement in the development of an immunocompetent human skin model, specifically tailored for investigating burn-induced innate or adaptive immune reactions at the site of burn injury.

## 1 Introduction

Burn injuries often trigger an excessive and uncontrolled immune response, resulting in various secondary complications such as systemic inflammation, delayed healing, wound deepening, and severe scarring (1–4). Gaining a better understanding of the underlying reactions responsible for burn-induced inflammation is crucial for effectively managing the inflammatory processes in burn wound healing. Detailed knowledge on the role and impact of specific immune cells and cytokines that are involved in the inflammatory response is, however, still limited. Most studies in this field rely on animal models, primarily rodents, which may not directly translate to the human context. To advance our understanding without relying on experimental animals, it is imperative to develop appropriate human 3D skin models that can study immune dysfunction at the site of burn injury.

Immediately after burn injury, pro-inflammatory neutrophils and macrophages accumulate at the wound site (5–7). These phagocytic cells play a critical role in eliminating cell debris and pathogens from the injured area (8). However, high numbers of hyperactive innate immune cells can damage healthy tissues and hamper the wound healing process (9–11). Initially, pro-inflammatory macrophages (referred to as M1) dominate, followed by anti-inflammatory macrophages (known as M2) in later stages, which suppress inflammatory responses and support wound healing (8, 12). Later in the inflammation process, T cells migrate to the wound area to coordinate targeted anti-pathogen responses and regulate ongoing inflammation to advance wound healing (13, 14). Different subsets of effector T cells, such as Th1 and Th17, are known to enhance inflammation, whereas Th2 and regulatory T cells (Tregs) are involved in the resolution of inflammation (15, 16). Achieving a proper immune balance between pro- and anti-inflammatory responses is critical for an uncomplicated and timely transition from inflammation to wound healing. Yet the exact mechanisms underlying distorted immune reactions after burn injury and the methods to restore proper immune function remain unclear.

Studying the immune response in burn patients faces limitations due to the absence of baseline measurements, inter-individual variability, differences in injuries, and constraints on collecting patient samples (6, 17). Consequently, most knowledge about the immune response following burn trauma is obtained from animal experiments (6, 17, 18) and has provided essential data for the advancement of human therapeutics. However, the use of experimental animals presents ethical concerns and translation challenges (19). Although valuable insights can be derived from animal studies, animals do not accurately reflect the human situation due to differences in skin architecture and wound healing processes (18, 20–22). It is therefore challenging to extrapolate relevant findings to burn patients. Thus, alternative approaches for research on burn wound healing need to be developed (23). *In vitro* human skin models are promising alternative experimental tools for studying various aspects of skin injury based on the behavior of keratinocytes and fibroblasts (23–25). Currently, many existing skin models fail to capture the complex processes of skin inflammation because they lack essential immune components (26, 27). In order to make *in vitro* skin models more useful and appropriate, it is necessary to incorporate immune cells and inflammatory mediators.

In this study, we aimed to develop a human full skin equivalent (FSE) based on the collagen-elastin matrix MatriDerm^®^ (28, 29), as we described previously (30). MatriDerm is a clinically applied matrix that provides a robust extracellular matrix architecture supporting skin regeneration in cutaneous defects (31–34). Our objective was to investigate the effect of burn injury on immune cells within the FSEs (1.13 cm^2^ with a burned surface of 19%). With a 19% burn, the effect of the injury is clearly present, while there is enough material and cells that is not destroyed so that cells can enter. We hypothesized that burn injury alters the inflammatory state of immune cells in these FSEs, which can be detected by changes in cell phenotype and cytokine expression. Cells from the innate and the adaptive immune system, namely monocytes and T cells, were isolated from human buffy coats and cultured in the FSE. We examined alterations in marker expression on immune cells and the secretion of cytokines in the culture medium.

## 2 Materials and methods

### 2.1 Human skin samples

Skin samples were obtained from adult patients who underwent abdominoplasty at the Red Cross Hospital in Beverwijk, Medical Clinic in Velsen or Spaarne Gasthuis in Haarlem. Samples from 17 different donors were used (donor age: 48 ± 13 years; sex: 93% female). Consent for the use of these anonymized, post-operative residual tissue samples was received through an informed opt-out protocol, in accordance with the national guidelines (https://www.coreon.org/) and approved by the institutional privacy officers. Subjects were actively informed of this procedure and were able to easily withdraw at any point. Split-thickness samples of 0.3 mm were harvested using a dermatome (Aesculap AG & Co. KG, Tuttlingen, Germany).

### 2.2 Isolation of human keratinocytes and fibroblasts

See **Supplementary Table 1** for the contents of culture media. Harvested skin was incubated in 0.25% dispase (Gibco, ThermoFisher Scientific, Paisley, UK) at 37°C for 45 min. The epidermis was separated from the dermis using forceps. For fibroblast isolation, the dermal part of the split skin was cut into small pieces and submerged into a 0.25% collagenase A (Roche, Basel, Switzerland) solution at 37°C for 2 h. After addition of 1 mM EDTA (Life Technologies, Paisley, UK) + PBS (Gibco) to inhibit enzyme activity, the cell suspension was poured through a 500 µm cell strainer (PluriSelect, Leipzich, Germany) and centrifuged for 10 min at 360 × *g*. The cell pellet was resuspended in culture medium and poured through a 70 µm cell strainer (Starstedt AG & Co. KG, Nümbrecht, Germany) and cultured at 37°C with 5% CO_2_. For keratinocyte isolation, the epidermis was transferred into 0.05% trypsin (Gibco) and incubated for 20 min at 37°C. The cell suspension was poured through a 70 µm cell strainer and centrifuged for 10 min at 110 × *g*. Next, the cell pellet was washed in culture medium and centrifuged for 10 min at 160 × *g*. The cell pellet was then resuspended in CnT-07 medium (CELLnTEC Advanced Cell Systems AG, Bern, Switzerland) and keratinocytes were transferred onto a 1 µg/cm^2^ collagen type IV (Sigma-Aldrich, Saint Louis, MO, USA)-coated culturing flasks (Starstedt) at 37°C with 5% CO_2_.

### 2.3 Human full skin equivalents

**Figure 1:**
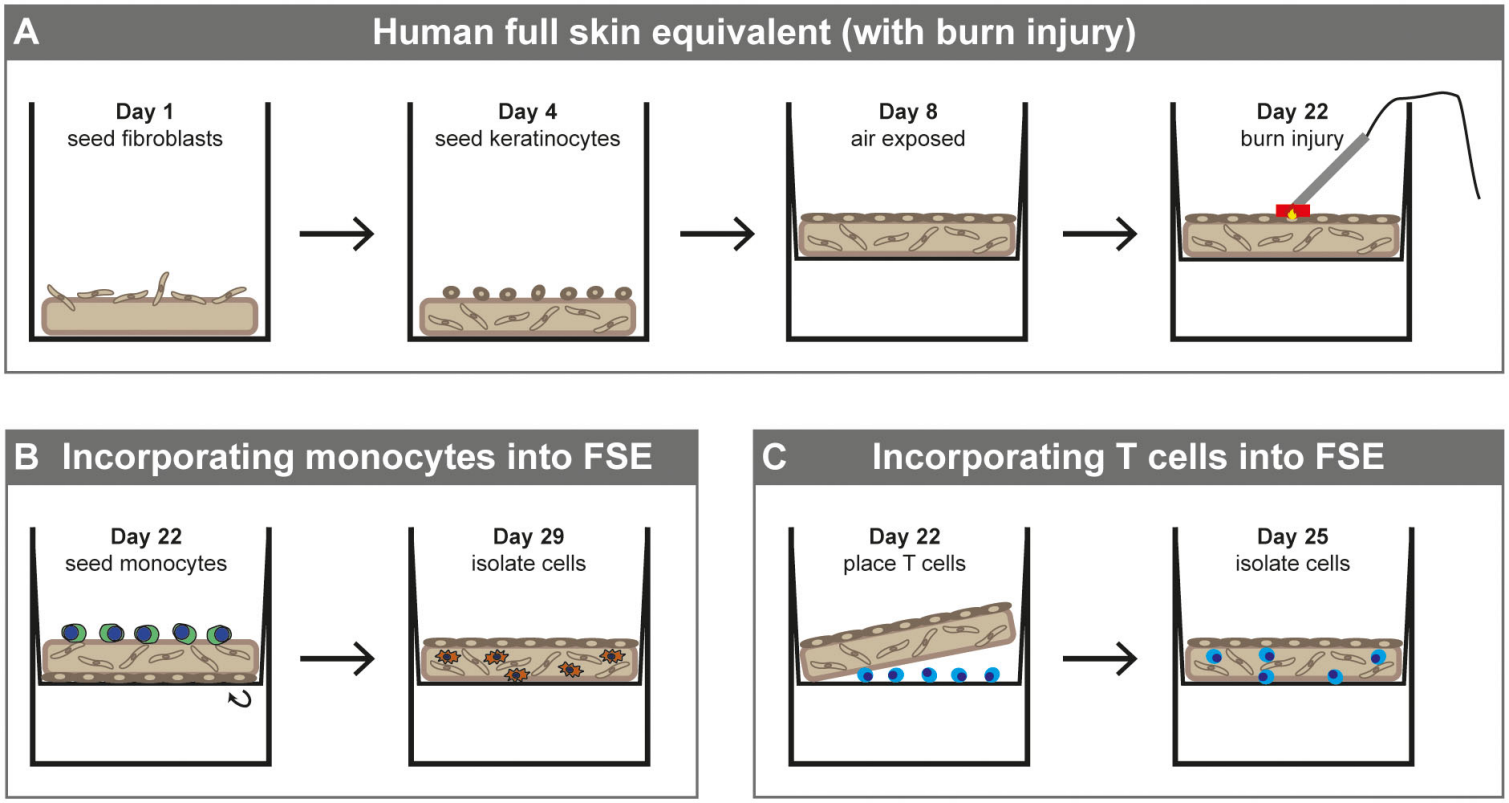
Development of human full skin equivalent (burn wound) model with monocytes or T cells. **(A)** Development of FSE. **(B)** Incorporating monocytes into FSE (directly after burn injury). **(C)** Incorporating T cells into FSE (directly after burn injury).

Our FSE development protocol was based on previous experiments (30). MatriDerm^®^ (MedSkin Solutions Dr. Suwelack AG, Billerbeck, Germany) with a thickness of 3 mm was cut into circular pieces of 1.13 cm^2^. At day one, 2 × 10^5^ fibroblasts were seeded onto the matrix and the matrix was submerged in culture medium containing 65 µg/mL ascorbic acid for 4 days at 37°C with 5% CO_2_ (**Figure 1A**). Subsequently, 1 × 10^5^ keratinocytes were seeded on the opposite side and the model was cultured submerged in FSE I medium containing 2 ng/ml KGF (ImmunoTools GmbH, Friesoythe, Germany) and 0.5 ng/ml EGF (R&D Systems, Inc., Minneapolis, MN, USA) for 4 days at 37°C with 5% CO_2_. Next, the FSE was transferred to a transwell (Starstedt) and cultured air-exposed in deep well plates (Greiner Bio-One BV, Alphen aan den Rijn, the Netherlands) with FSE II medium containing 4 ng/ml KGF and 1 ng/ml EGF. From day 11 onward the FSE was cultured in FSE III medium containing 4 ng/ml KGF and 1 ng/ml EGF and from day 15 onward in FSE III medium that was refreshed twice weekly. At day 22, the FSE was ready to use for immune cell culture. Cell numbers and culture conditions are based on preceding experiments (30).

### 2.4 Induction of burn injury

Our burn injury procedure was based on previous experiments (30). A copper plate (2 × 10 mm) attached to a PACE intelliHeat ST50 soldering iron (Vass, USA) was heated to 80-90°C and stably applied to the epidermal side of the FSE for 20 sec to make contact without exerting pressure or indenting the FSE samples (**Figure 1A**). The temperature of the copper device was measured by an external digital thermometer (Farnell InOne, Utrecht, the Netherlands). Using this procedure, we created a burn injury that covered about 19% of the surface area of the model.

### 2.5 PBMC isolation from human buffy coat

PBMCs were isolated from buffy coats obtained from healthy donors (Sanquin, Amsterdam, the Netherlands) by density gradient centrifugation using Lymphoprep (Stemcell Technologies, Vancouver, Canada). The buffy coat was diluted in 0.5% Bovine serum albumin in PBS and layered over the density gradient medium. After centrifugation at 1000 × *g* for 15 min (without brakes), the PBMCs were collected in FSE I medium. Cells were resuspended in 50% fetal bovine serum (Gibco) + 40% FSE I medium + 10% dimethyl sulfoxide. After 24 h storage in Mr. Frosty (ThermoFisher scientific) with isopropanol at -80°C, cells were stored in liquid nitrogen until use.

### 2.6 Incorporating monocytes into the FSE

PBMCs were incubated with anti-CD14 beads (Invitrogen, Waltham, MA, USA) at a bead/cell ratio of 2.5:1 at 2-8°C for 20 min on a tube roller. Monocytes were isolated from the PBMCs using a magnet (Invitrogen Dynal AS, Oslo, Norway). Monocytes were resuspended in FSE I medium and 2.5 × 10^5^ cells were added to the dermal side of the FSE. For burn-injured FSEs, the cells were added directly after burn injury was inflicted. Inverted FSE with monocytes was incubated at 37°C for 2 h and subsequently placed back into the transwell (**Figure 1B**). The FSE with monocytes was cultured for 7 more days with a medium change at day 3.

### 2.7 Incorporating T cells into the FSE

Lymphocytes were isolated by culturing PBMCs in a culture flask. After 24 h, adherent cells were removed. T cells were activated by adding anti-CD3/CD28 Dynabeads (Gibco) at a bead/cell ratio of 5:1 at 37°C for 4 h. After the activation, cells were resuspended in FSE I medium and 2.5 × 10^5^ cells were placed between the transwell membrane and the dermal side of the FSE (**Figure 1C**), based on previous findings (35). Of these cells, 71 ± 14% was CD3^+^. For burn-injured FSEs, the cells were added directly after burn injury was inflicted. The FSE with T cells was cultured for 3 more days.

### 2.8 Dissociation of FSE for flow cytometry analysis

The FSE dissociation procedure was based on a protocol from He et al. (36). Macrophage FSEs were incubated with 0.25 U/ml collagenase A (Roche) at 37°C in a shaking water bath for 20 min. Because enzymes affect the expression chemokine receptors (37), T cell models were not dissociated using collagenase A. FSEs were then put in C-tubes (Miltenyi Biotec GmbH, Bergisch Gladbach, Germany) with 5 mL of PBS containing 1 mM EDTA and (further) dissociated by running program “B” twice on a tissue dissociator (gentleMACS, Miltenyi Biotec GmbH). Samples were passed through a 500 µm cell-strainer (PluriSelect) and then a 40 µm cell strainer (Sarstedt) to obtain a single cell suspension.

### 2.9 Flow cytometry

Single cell suspensions were stained using the macrophage or T cell panel (**Supplementary Table 2**). Zombie Aqua (BioLegend, San Diego, CA, USA) was used in the macrophage panel and propidium iodide (Miltenyi Biotec GmbH) was used in the T cell panel to determine viability of cells. Stained cell samples were acquired on the flow cytometer (MACS Quant Analyzer 10, Miltenyi Biotec GmbH) and gating (**Supplementary Figure 1**) was performed in FlowLogic (Inivai Technologies, Victoria, Australia).

### 2.10 Immunohistochemistry

See **Supplementary Table 3** for antigen retrieval and primary antibodies. Kryofix (50% ethanol + 7% PEG300 in demineralized water)-fixed paraffin-embedded samples were cut into sections with a thickness of 5 µm and rehydrated followed by hematoxylin and eosin staining or blocking of endogenous peroxidase using 1% hydrogen peroxide at room temperature for 15 min. After antigen retrieval was performed, sections were pre-incubated with 5% normal goat serum (Sigma-Aldrich) diluted in PBS + 1% bovine serum albumin (ThermoFisher). Sections were then incubated with primary antibodies at room temperature for 1 h followed by incubation with a poly-HRP-goat-anti-mouse or rabbit secondary antibody (BrightVision, VWR, Amsterdam, the Netherlands) at room temperature for 30 min. After washing, detection was established using 3,3′-diaminobenzidine (DAB). After DAB staining was completed, sections were counterstained with hematoxylin, dehydrated and mounted with Eukit Mounting Medium (Sigma-Aldrich).

### 2.11 Lactate dehydrogenase staining

Snap-frozen FSEs from -80°C were thawed and fixated in 1% paraformaldehyde (Sigma) for 2 h at 4°C. The FSEs were then put in 20% sucrose (Sigma) in PBS solution overnight at 4°C. FSEs were embedded in Tissue Tek OCT (Sakura Finetek Europe B.V., Alphen aan de Rijn, Netherlands) and sections of 10 µm were cut using a cryotome (Slee MNT, Adamas Instruments B.V., Rhenen, Netherlands). Dried sections were washed in PBS and incubated with LDH solution (2 mM Gly-Gly (Sigma); 0.75% NaCl (Sigma); 5% polypep (Sigma); 1.75 mg/ml β-nicotinamide adenine dinucleotide (Sigma); 3 mg/ml nitroblue tetrazolium (Sigma) in demineralized water of pH 8) for 3 h at 37°C. Sections were washed in tap water at 50°C and in PBS and then stained with Eosin Y for 4 min. Sections were then put in PBS for 1 sec, acetone for 30 sec, acetone/xylene (1:1) for 1 min and xylene for 1 min, before embedding with Eukit Mounting Medium.

### 2.12 Microscopy

Microscopic visualization was performed with a Zeiss Axioskop40FL microscope (Zeiss, Breda, The Netherlands). Images were acquired using a Nikon Eclipse TS2 camera and the NIS-Elements software version 4.4 (Nikon Instruments, Amsterdam, The Netherlands).

### 2.13 Re-epithelization rate

Length of re-epithelization in the FSEs was measured in microscopic images of H&E-stained sections by two assessors using NIS-Elements software. The mean of the two wound sides was used for analysis.

### 2.14 Immunoassay

Cytokines, chemokines and growth factors were analyzed in samples of medium. Neat samples were measured using the Human Essential Immune Response LegendPlex Multi-analyte Flow Assay kit (cat. 740929, BioLegend), according to the manufacturer’s instructions and were acquired on the flow cytometer. This 13-plex immunoassay included: IFN-γ, IL-1β, IL-2, IL-4, IL-6, IL-8 (CXCL8), IL-10, IL-12p70, IL-17A, IP-10 (CXCL10), MCP-1 (CCL2), TNF-α and TGF-β1. Concentrations were determined using FlowLogic software. When cytokine levels were lower than the standard range, the lowest level of quantification was used. When cytokine levels were higher than the standard range, the levels were estimated based on the fluorescent signal in the assay.

### 2.15 Statistical analysis and data visualization

We used the Shapiro-Wilk test in R (ggpubr and ggplot2 packages, open source) to determine distribution of data and found that the majority of data were not normally distributed. Therefore, differences in cell number/percentages and cytokines levels between different modeling conditions were explored using Mann-Whitney U test in R (ggpubr and ggplot2 packages, open source). Data was visualized using R (ggplot2 package, open source) and significant (p value of < 0.05) differences were indicated by asterisks.

## 3 Results

### 3.1 Human FSEs facilitate the study of burn injury *in vitro*

**Figure 2:**
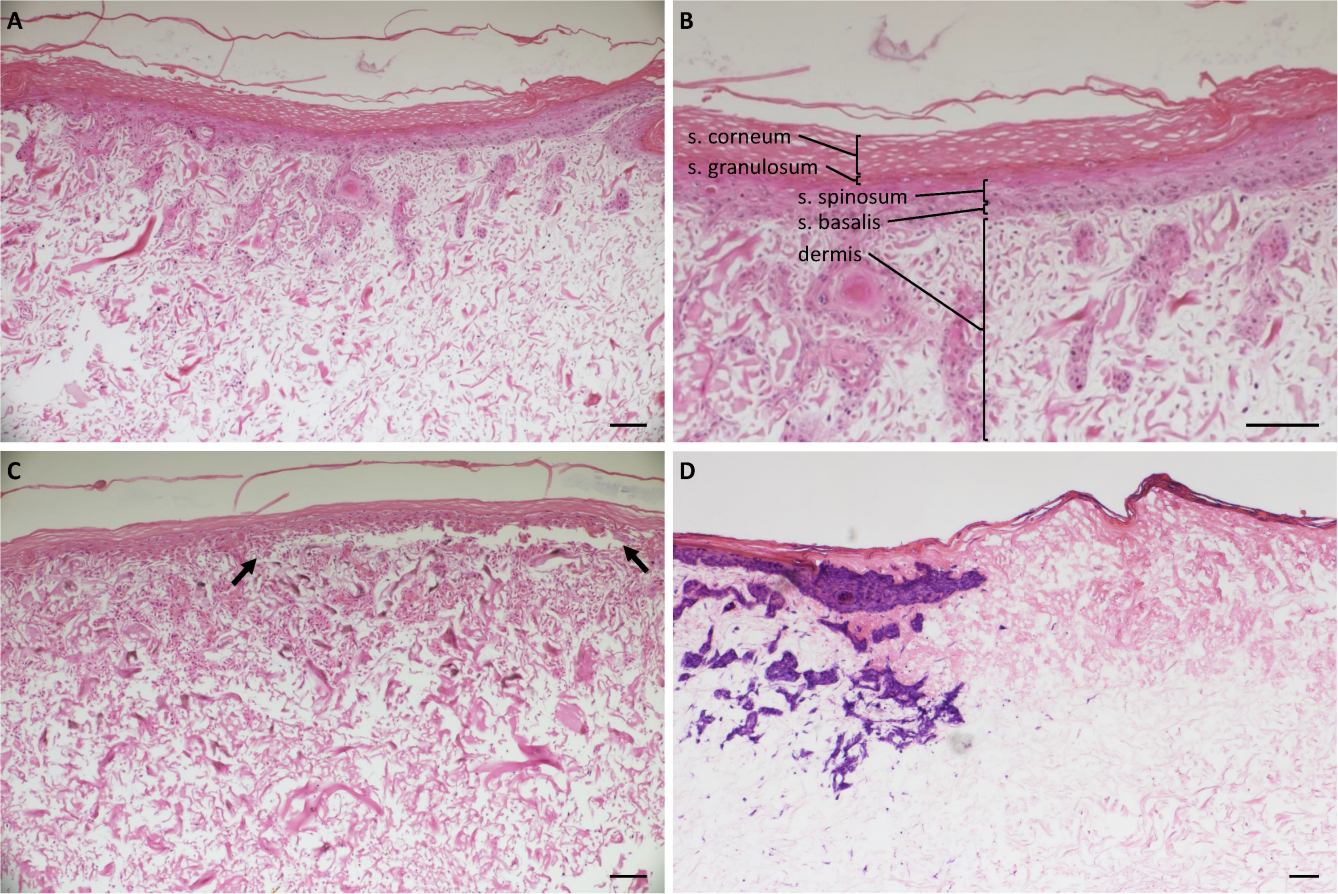
Histology of uninjured and burn-injured FSEs. H&E staining of **(A)** Uninjured FSE after 3 weeks of culture; **(B)** Uninjured FSE after 3 weeks of cultured at a higher magnification. Dermis and the different epidermal layers are indicated: stratum corneum, s. granulosum, s. spinosum and s. basalis; **(C)** FSE after 3 weeks of culture and 3 days after burn injury. The detached epidermis caused by the burn is clearly visible between black arrows. **(D)** Immunohistochemical LDH staining of an FSE after 3 weeks of culture and 3 days after burn injury. Blue-purple staining indicates viable cells present in the epidermis (keratinocytes) and dermis (fibroblasts) up until the wound edge. Experiments were performed in duplicate using keratinocytes and fibroblasts from 6 different donors. Black scale bar = 100 µm; black arrows indicate burn injured area.

FSEs were generated by seeding human keratinocytes and fibroblasts into a collagen-elastin containing matrix from MatriDerm (see **Figure 1A** for procedure), as we described previously (30). After 3 weeks of culture, the FSEs presented a well-established epidermis and dermis (**Figures 2A, B**). Burn injury inflicted on the FSE was visualized by microscopy. Three days post injury the burn wound was visible, characterized by detachment of the epidermis from the affected region of the dermis (**Figure 2C**). Staining for lactate dehydrogenase (LDH) (38) showed viable cells in the dermis (fibroblasts) and epidermis (keratinocytes) up to the wound edge but not in the wound, confirming that the injury resulted in cell damage (**Figure 2D**).

### 3.2 Monocytes differentiated into macrophages in the FSEs and showed upregulated M1 marker expression upon burn injury

**Figure 3:**
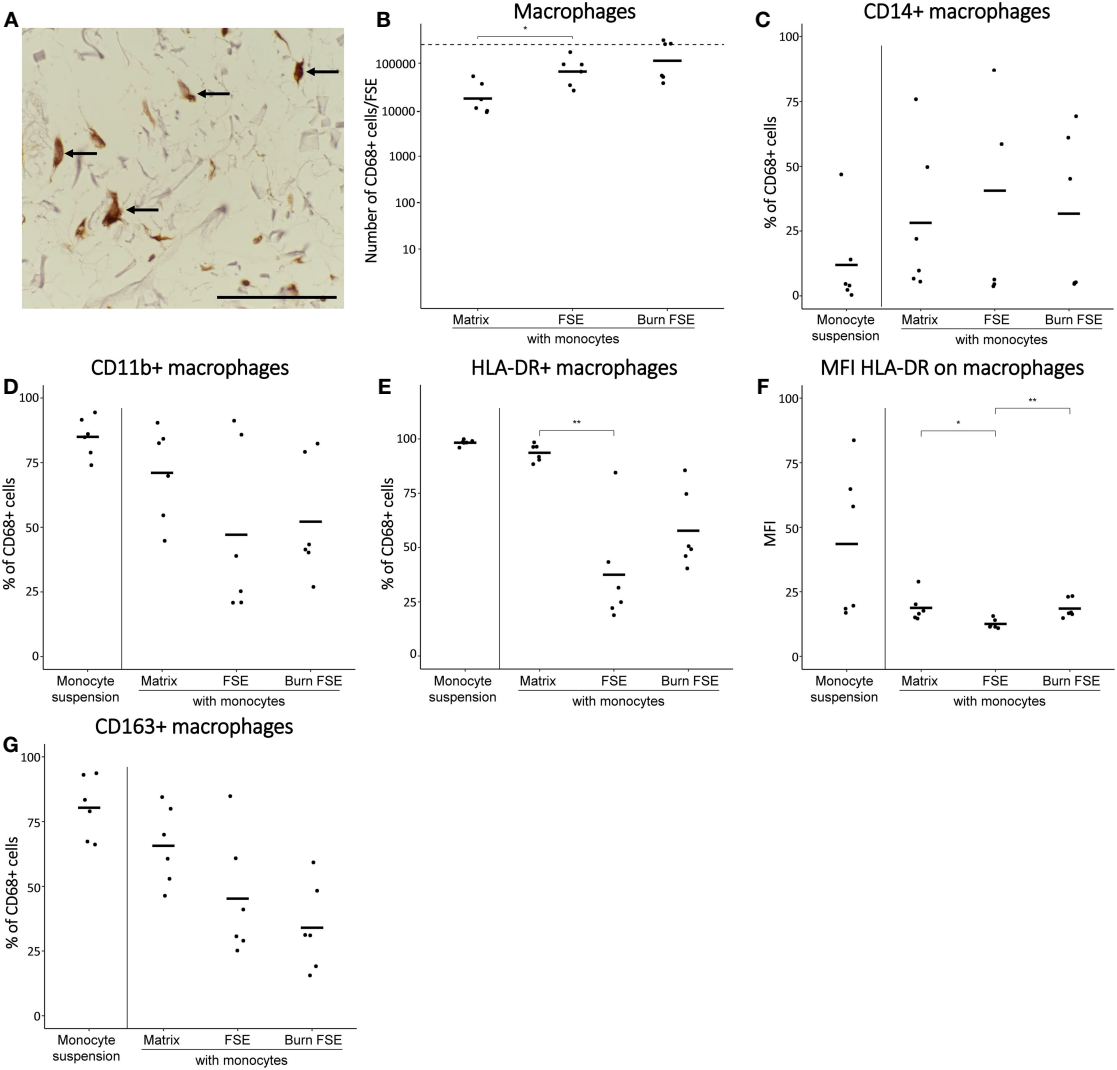
Monocytes after 7 days of culture in (burn-injured) FSEs. **(A)** Immunohistochemical CD68 staining of an injured FSE. Black arrows point to positive cells in the FSE. **(B)** Number of CD68^+^ cells (macrophages) per FSE after isolation based on flow cytometry; dashed line indicates the number of monocytes added to the dermal side of the FSE. Percentage of CD68^+^ cells (macrophages) that were **(C)** CD14^+^; **(D)** CD11b^+^; **(E)** HLA-DR^+^. **(F)** Mean fluorescence intensity (MFI) of HLA-DR on CD68^+^ cells macrophages. **(G)** Percentage of CD68^+^ cells (macrophages) that were CD163^+^. Experiments were performed in duplicate using 6 different keratinocyte donors, 6 fibroblast donors and 4 monocytes donors. Only comparisons between monocytes in matrix, in uninjured FSEs and in burn-injured FSEs are shown. Statistically significant differences were calculated using Mann-Whitney U test. Significant differences are indicated by asterisks: *p < 0.05; **p < 0.01.

Unstimulated monocytes were introduced to full-established (burn-injured) FSEs to simulate an innate immune response. To prevent the cells from adhering to the transwell membrane, monocytes (about 2.5 × 10^5^) were administered directly to the dermal side of the FSEs (see **Figure 1B** for procedure). Monocytes cultured in suspension or in matrix without skin cells served as controls. Through microscopy analysis, we confirmed the presence of monocytes within the FSE in both uninjured and burn-injured models (**Figure 3A**). Monocytes seemed to downregulate or lose monocyte marker CD14 (data not shown) and upregulate the expression of macrophage marker CD68 in the cultured FSEs, regardless of burn injury (**Figure 3A**). This showed that the monocytes differentiated into macrophages within a span of 7 days. Rate of re-epithelization in the FSEs after 7 days was 347 ± 168 µm. The re-epithelization rate was slightly higher in FSEs with monocytes (439 ± 126 µm), but did not reach significance.

To study the effect of burn injury on these monocyte-derived macrophages in more detail, FSEs were dissociated after 7 days of culturing the full-established FSEs. Using flow cytometry, we identified the macrophages based on their expression of CD68 (macrophage marker), CD14 (monocyte marker), CD11b (activation marker), HLA-DR (M1 differentiation marker) and CD163 (M2 differentiation marker). In uninjured FSEs, an average of 8.0 ×10^4^ CD68^+^ macrophages were present (**Figure 3B**). There was high variability in the fraction of CD68^+^ macrophages that expressed CD14 or CD11b (**Figures 3C, D**), irrespective of burn injury. This variation in macrophage differentiation and activation within the FSEs, was presumably dependent on the donor (buffy coat, fibroblast or keratinocyte donor). Comparing the FSEs to macrophages cultured in the matrix without skin cells, we observed a smaller proportion of HLA-DR^+^ or CD163^+^ (**Figures 3E, G**) macrophages in the FSEs. Burn injury appeared to increase the average number of CD68^+^ macrophages in the FSE (1.6 × 10^5^; **Figure 3B**), although not significantly. Interestingly, the percentage of CD14^+^ macrophages was significantly decreased after burn injury (**Figure 3C**). Furthermore, burn injury significantly increased the expression of HLA-DR on macrophages (**Figure 3F**) and appeared to decrease the percentage of CD163^+^ macrophages within the FSEs (**Figure 3G**). Thus, we generated a human FSE model incorporating monocytes capable of actively differentiating into macrophages during culture and observed that burn injury appeared to enhance M1 differentiation of macrophages.

### 3.3 Inclusion of monocytes in FSEs slightly increased production of inflammatory cytokines, regardless of burn injury

**Figure 4:**
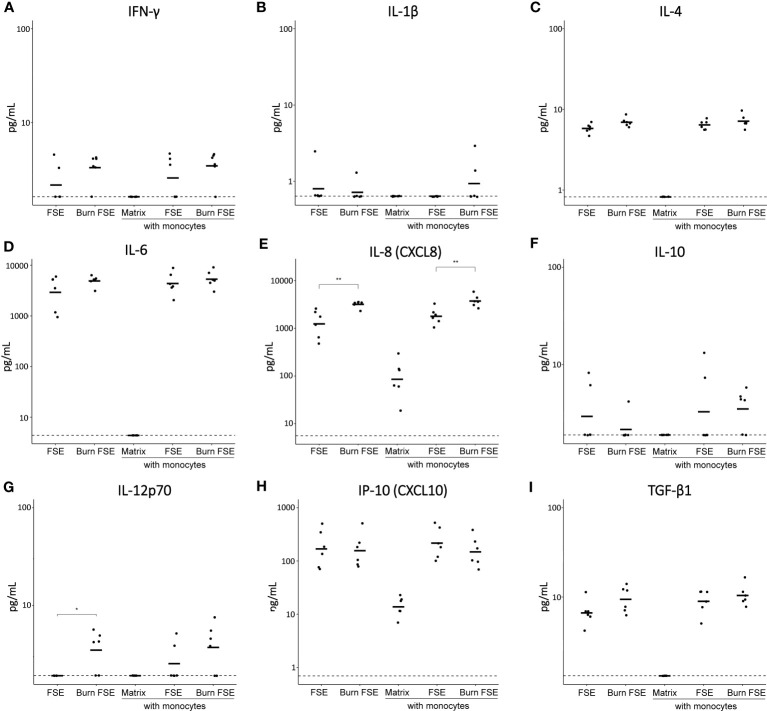
Cytokine levels in medium of (burn-injured) FSEs after 7 days of culture with monocytes. **(A)** IFN-γ; **(B)** IL-1β; **(C)** IL-4; **(D)** IL-6; **(E)** IL-8; **(F)** IL-10; **(G)** IL-12p70; **(H)** IP-10; **(I)** TGF-β1. Samples from biological duplicates were averaged per donor. Concentrations are reported in pg/mL medium. Experiments were performed in duplicate using 6 different keratinocyte donors, 6 fibroblast donors and 4 monocytes donors. The dashed line indicates the lowest level of quantification. Statistically significant differences were calculated using Mann-Whitney U test. Only comparisons between uninjured and burn-injured models or between models without and with monocytes are shown. Significant differences are indicated by asterisks: *p < 0.05; **p < 0.01.

At day 7 (when FSEs were terminated), the levels of 13 inflammatory cytokines in the culture media were analyzed (**Figure 4**). In the absence of monocytes, FSEs secreted high levels of IL-6, IL-8 and MCP-1 (**Figure 4D, E**; **Supplementary Figure 2**). Burn injury significantly increased the level of IL-8 and IL-12p70 (**Figures 4E, G**). Because of the high MCP-1 levels in the FSEs, the cytokine assay reached maximum signals, making it impossible to detect differences in MCP-1 levels between burn-injured and uninjured models. When monocytes were incorporated into the FSEs, there was a slight increase in the levels of IL-4, IL-6, IL-8, IP-10 and TGF-β1 (**Figures 4C-E, H, I**). Burn injury on the monocyte incorporated FSEs led to a further increase of IL-8. Cytokines IL-2, IL-17A and TNF-α were not detected in any of the experimental conditions (**Supplementary Figure 2**).

### 3.4 T cells that migrated into FSEs expressed Th1 and Th17 chemokine receptors, irrespective of burn injury

**Figure 5:**
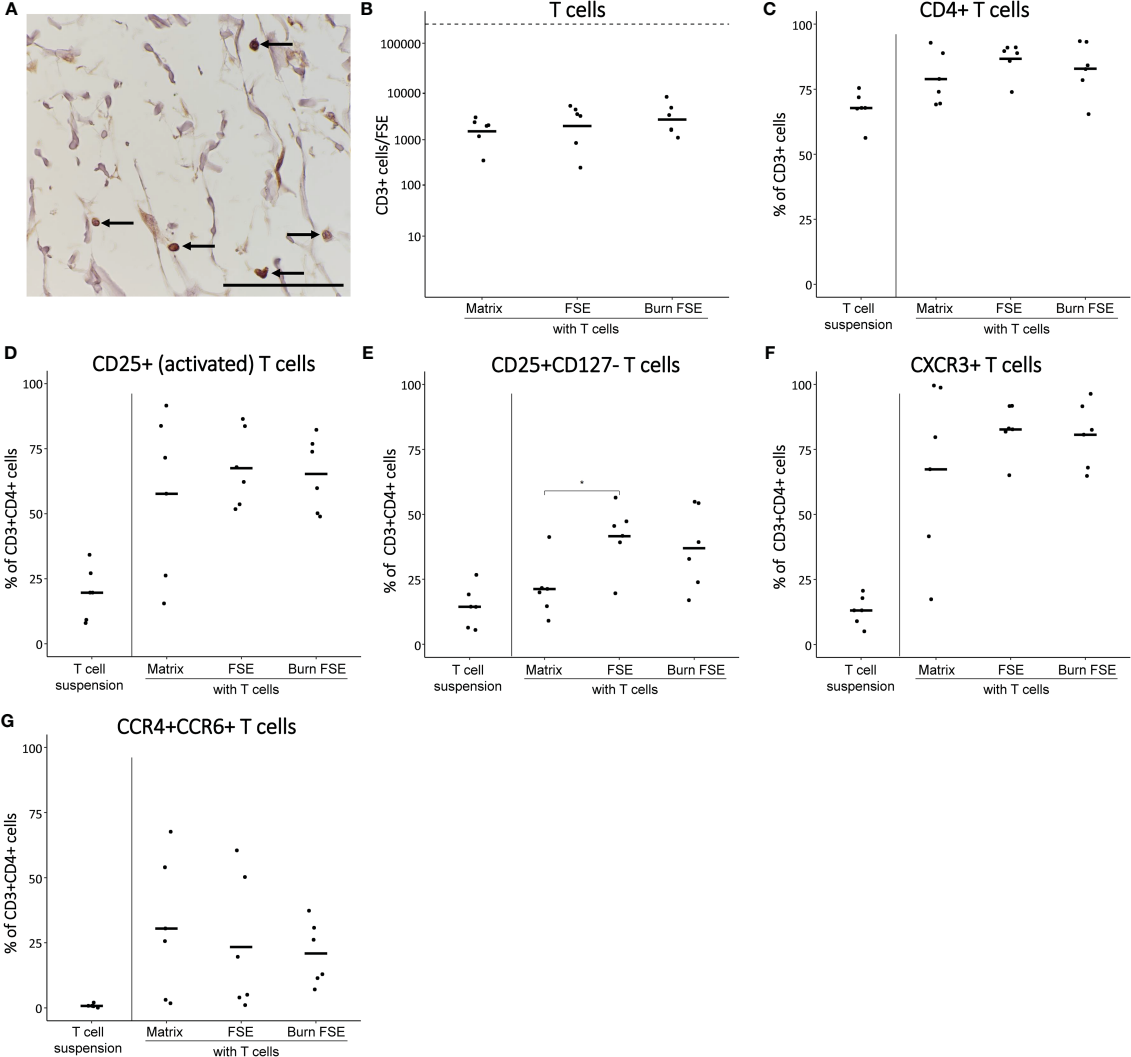
Pre-activated T cells after 3 days of culture in (burn-injured) FSEs. **(A)** Immunohistochemical CD3 staining of an injured FSE. Black arrows point to positive cells in the FSE. **(B)** Number of T cells (CD3^+^ cells) per FSE after isolation using flow cytometry; dashed line indicates the number of T cells added to the transwell. **(C)** Percentage of CD3^+^ (T cells) that are CD4^+^. Percentage of CD3^+^CD4^+^ T cells that were **(D)** CD25^+^; **(E)** CD25^+^CD127¯; **(F)** CXCR3^+^; **(G)** CCR4^+^CCR6^+^. Experiments were performed in duplicate using 6 different keratinocyte donors, 6 fibroblast donors and 5 T cell donors. Only comparisons between T cells in matrix, in uninjured FSEs and in burn-injured FSEs are shown. Statistically significant differences were calculated using Mann-Whitney U test. Significant differences are indicated by asterisks: *p < 0.05.

To simulate an adaptive immune response, we introduced CD3/CD28 bead pre-activated T cells into fully-established (burn-injured) FSEs. Approximately 2.5 × 10^5^ T cells were placed between the transwell membrane and the dermal side of the FSEs, and they were cultured for a duration of 3 days (see **Figure 1C** for procedure), following a previously established protocol (35). Pre-activated T cells cultured in suspension or in matrix without skin cells served as controls. Using microscopy, we could detect CD3^+^ T cells that had actively migrated into the FSEs (**Figure 5A**). As 3 days was too soon after burn injury, the re-epithelization rate in these FSEs could not be measured.

Following a 3-day culture period, FSEs were dissociated to perform flow cytometric analysis of T cells. T cell differentiation was examined based on their expression of CD3 (T cell marker), CD4 (effector T cell marker), CD25/CD127 (activation marker and regulatory T cell marker), CXCR3 (Th1 differentiation marker) and CCR4/CCR6 (Th17 differentiation marker). Only a small portion (2.8 × 10^3^) of T cells had migrated into the FSEs (**Figure 5B**). Among these migrated T cells, the majority (approximately 86.7%) were CD4^+^ T cells (**Figure 5C**). Most of these CD4^+^ T cells expressed CD25, indicating their activation and suggesting a correlation between T cell activation and migration (**Figure 5D**). The percentage of CD25^+^CD127¯ T cells, potentially indicating Treg differentiation, was higher in the FSEs compared to T cells cultured in the matrix alone (**Figure 5E**). Furthermore, the FSEs contained a higher percentage of CXCR3^+^ T cells, indicating enhanced Th1 activity (**Figure 5F**). Similarly, an increase in the percentage of CCR4^+^CCR6^+^ T cells was observed in the FSEs, suggesting augmented Th17 activity (**Figure 5G**). The average number of T cells in burn-injured FSEs was comparable to that in the uninjured FSEs (**Figure 5B**) and burn injury did significantly not affect the investigated T cell markers (**Figures 5D-G**). Together, our findings demonstrate that particularly activated T cells migrated into the FSEs, and there is a potential enhancement of Treg and Th1/Th17 activation, regardless of burn injury.

### 3.5 Inclusion of T cells in uninjured or burn-injured FSEs increased the levels of inflammatory cytokines

**Figure 6:**
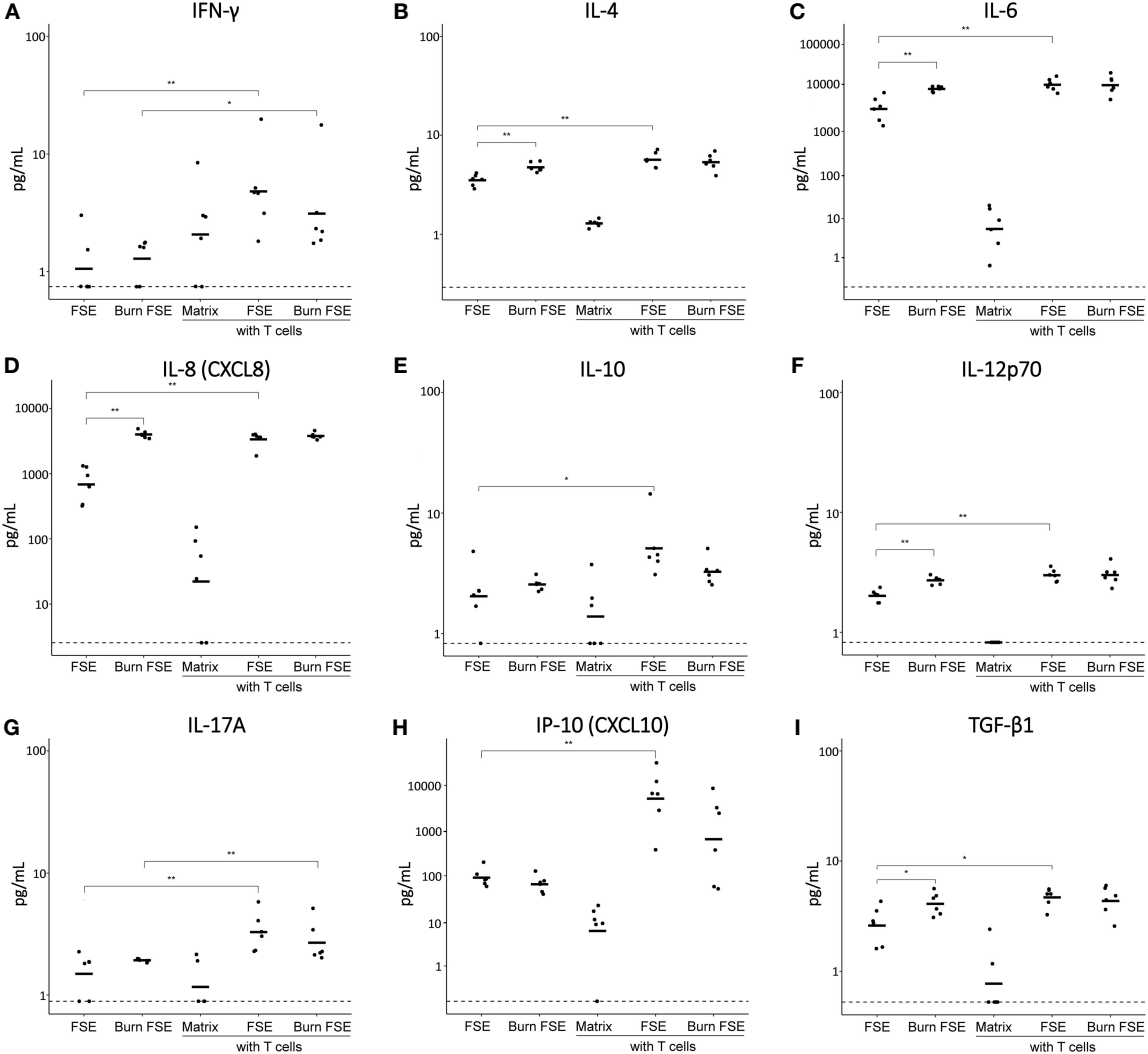
Cytokine levels in medium of (burn-injured) FSEs after 3 days of culture with pre-activated T cells. **(A)** IFN-γ; **(B)** IL-4; **(C)** IL-6; **(D)** IL-8; **(E)** IL-10; **(F)** IL-12p70; **(G)** IL-17A; **(H)** IP-10; **(I)** TGF-β1. Samples from biological duplicates were averaged per donor. Concentrations are reported in pg/mL medium. Experiments were performed in duplicate using 6 different keratinocyte donors, 6 fibroblast donors and 5 T cell donors. The dashed line indicates the lowest level of quantification. Statistically significant differences were calculated using Mann-Whitney U test. Only comparisons between uninjured and burn-injured models or models without and with T cells are shown. Significant differences are indicated by asterisks: *p < 0.05; **p < 0.01.

To investigate cytokine secretion in the T cell-incorporated FSEs, we analyzed the culture medium at day 3. FSEs without T cells produced high levels of IL-6, IL-8 and MCP-1 (**Figures 6C, D**; **Supplementary Figure 3**), consistent with the expression observed after 7 days of culture (**Figures 4C, D**; **Supplementary Figure 2**). In FSEs cultured without T cells, burn injury significantly increased the levels of IL-4, IL-6, IL-8, IL-12p70 and TGF-β1 (**Figures 6B-D, F, I**).

Introducing T cells into uninjured FSEs resulted in elevated levels of IFN-γ, IL-2, IL-4, IL-6, IL-8, IL-10, IL-12p70, IL-17A, IP-10 and TGF-β1 (**Figures 6A-I**; **Supplementary Figure 3**). While burn injury did not further increase the levels of these cytokines, it slightly decreased the levels of IL-10 and IP-10 in the presence of T cells. IL-2 was only detected in the presence of T cells and no significant differences were observed for the levels of IL-1β and TNF-α (**Supplementary Figure 3**). Overall, the inclusion of T cells in the FSEs appeared to further increase both pro- and anti-inflammatory cytokines, while burn injury specifically reduced the T cell induced levels of IL-10 and IP-10.

## 4 Discussion

There is a pressing need for appropriate, animal-free models to investigate immune reactions following burn injury. Conventional FSEs cannot capture the complex immune responses associated with burn injury because they lack crucial immune components such as monocytes and T cells (5, 26, 27, 30, 39–43). As monocytes are actively involved in the acute inflammatory phase and T cells are crucial for regulation of ongoing inflammation, these cells are essential to model the burn immune response more accurately (44, 45). In this study, we developed an FSE and incorporated monocytes or T cells to simulate innate and adaptive immune reactions to burn injury, respectively. Flow cytometry analysis of human primary monocytes or T cells cultured in the FSEs allowed us to examine changes in immune cell phenotype and cytokine expression between 3 to 7 days.

Certain cytokines, namely IL-6, IL-8 and MCP-1, were expressed by the FSEs even in the absence of immune cells. This secretion of cytokines was also seen by others (40) and likely originates from stress responses in fibroblasts and keratinocytes induced by *in vitro* culturing and skin morphogenesis. This cell stress response should not be overlooked; however, information regarding its cause or methods to reduce it is very limited. Interestingly, burn injury further increased the levels of IL-4, IL-6, IL-8, IL-12p70 and TGF-β1, significantly at day 3. By day 7, only IL-8 and IL-12p70 remained significantly increased compared to uninjured FSEs, suggesting a reaction of the fibroblasts and/or keratinocytes to the burn injury. This indicates that these cytokines are likely involved in the initiation of an inflammatory response. Previous studies utilizing fibroblasts and keratinocytes in similar collagen matrices have also reported increased levels of pro-inflammatory cytokines, such as IL-6, IL-8, and MCP-1, in response to burn injury (30, 40, 46).

We demonstrated the differentiation of monocytes into macrophages in these FSE within 7 days. This was shown by upregulation of CD68 expression in monocytes, consistent with previous findings by Smith et al. and Safi et al. (47, 48). Burn-injured FSEs contained macrophages with enhanced expression of HLA-DR compared to uninjured FSEs, indicating an M1-like response of macrophages to burn injury. HLA-DR expression on macrophages is an MHC class II molecule associated with inflammatory stimuli and M1 activity (49). The high percentage of HLA-DR^+^ macrophages that we found when they were cultured in suspension could be attributed to the culture conditions such as the media or the cell repellent surface (50). Although CD163 expression, indicative of M2 activation, showed a slight decrease in burn-injured FSEs, it was not statistically significant. Variation in the number of cells expressing CD14 and CD11b markers among different PBMC donors suggests distinct (donor-dependent) activation or differentiation rates. Although the increase of M1 macrophages is advantageous early during wound healing, it might slow down wound healing when M1 macrophages persist in the wound area. In this study, we did not observe a significant difference in re-epithelization rate between FSEs with or without macrophages. It would be interesting to see how these macrophages behave over a longer period of time and if they can be manipulated towards M1 or M2 to either delay or accelerate wound healing, as is suggested to happen *in vivo* (20). Despite the increased expression of HLA-DR on macrophages, there was a minimal effect on cytokine expression. In order to observe an effect on cytokine expression, the model might need to include higher numbers of monocytes or specific macrophage subtypes.

Several studies have developed skin models with macrophages to investigate skin diseases such as inflammatory skin disorders and carcinoma. For instance, Chung et al. co-cultured FSEs with RAW264.7 cells to simulate inflammatory skin responses, highlighting interactions between skin cells and macrophages that affect cytokine production and the degree of inflammation (51). In this model, the FSE was placed on a transwell membrane while RAW264.7 cells were cultured underneath the transwell. Linde et al. developed a human skin squamous cell carcinoma model incorporating PBMC-derived macrophages to study macrophage polarization and identified M2 activation in their tumor model (52). In another study, Bechetoille et al. produced a dermal construct with fibroblasts and investigated the effect of introducing dermal-type macrophages on cytokine production and macrophage phagocytic potential (53). Our study uniquely focused on the effect of burn injury on primary monocytes within a 3D skin model, allowing flow cytometry and cytokine production analysis.

When pre-activated T cells were introduced to the FSEs, a fraction of these cells actively migrated into the FSEs. The population of migrated T cells showed increased numbers of both Th1 receptor CXCR3 expressing cells (54) and Th17 receptors CCR4/CCR6 expressing cells (55), regardless of burn injury. This coincided with elevated levels of pro-inflammatory cytokines such as IFN-γ, IL-6, IL-8, IL-12p70, IL-17A, and IP-10. The production of chemokines like IP-10, induced by IFN-γ, is known to occur in inflamed tissue (56, 57). IP-10 is a chemoattractant for T cells and binds to chemokine receptor CXCR3 (58). The decrease in IP-10 production in burn-injured FSEs may be attributed to the loss of keratinocytes caused by the burn injury, as about 19% of the surface area of the model was burned. Moreover, the percentage of CD25^+^CD127¯ T cells, possibly Tregs, was increased, accompanied by elevated levels of IL-4, IL-10, and TGF-β1. However, IL-10 production was slightly reduced in burn-injured FSEs, which could be related to keratinocyte destruction or impaired regulatory activity caused by burn injury. Nevertheless, more research is needed to elucidate the role of different T cells during wound healing.

Our approach to incorporate T cells into the FSEs was inspired by previous studies in which T cells were cultured in an epidermal construct to examine their interactions with keratinocytes (35, 59). Similar skin models have been utilized to understand the pathophysiology of skin diseases such as psoriasis or atopic dermatitis (35, 60, 61). In these studies, T cells were stimulated to favor Th1/Th17 responses to explore their role in psoriatic skin models (60, 61). Shin et al. established a T cell model that showed a psoriatic epidermal phenotype and characteristic cytokine profiles and responded to various classes of psoriasis drugs (60). After infiltration of activated T cells, the psoriatic skin model from Lorthois et al. displayed a strong psoriasis-like activated inflammatory phenotype, including altered differentiation of keratinocytes and increased secretion of pro-inflammatory cytokines (61).

In our study, only a small fraction of pre-activated T cells migrated into the FSEs. Several factors may have contributed to this limited migration, including incomplete activation or overactivation of T cells, T cell death, insufficient migratory activity, or suboptimal isolation of the cells from the FSEs. To preserve the presence of chemokine receptors, we performed the isolation of T cells from FSEs without the use of collagenase, which is known to affect these receptors. However, this approach might have led to a lower yield of T cells compared to monocytes/macrophages obtained from the FSEs. The migratory activity of T cells can be enhanced by introducing additional chemotactic stimuli, such as T cell chemokines MIP-1α (CCL3), MIP-1β (CCL4), and RANTES (CCL5) (44). Exploring the effects of prolonged culture on the migratory activity as well as the phenotype and cytokine production of T cells would also be of interest. Furthermore, the technique used to prepare T cells can be improved by using magnetic or fluorescence cell sorting to establish an enriched population of T cells prior to their introduction into the model.

Our FSE model offers distinct advantages over other models by utilizing primary cells rather than cell lines, thereby making these models more representative for the *in vivo* situation. Furthermore, unlike microscopy-based studies, our research employed flow cytometry for quantification and analysis of macrophages and T cells. Although our flow cytometry panel did not include markers specific to keratinocytes and fibroblasts, investigating their expression of markers such as elafin, CK10, CK17, CD10, Ki67, FAP, or α-SMA, could provide more detailed insights into the effect of monocytes or T cells on burn wound healing processes. Although the effect of burn injury on the studied monocyte and T cell markers appeared limited in our current set-up, further investigations involving other time points, longer culture periods, distinct immune cell activation methods or different burn techniques (i.e. burn temperature or duration) are warranted. In addition, our model can be used to study the effect of burn injury on specific immune cell subsets, or a combination thereof. This can be achieved by differentiating monocytes into M1 or M2 macrophages or skewing T cells towards Tregs, Th1, Th2, or Th17 cells before introducing them into the FSEs. Including neutrophils in the model, despite the challenges involved, would also contribute to a better understanding of the burn-induced immune response and their role in wound healing (5, 62). However, culturing neutrophils in FSEs is difficult due to their short lifespan and the inability to cryopreserve them (63). Integrating a combination of certain immune cell subsets in the FSE will create an even more realistic environment to simulate burn wound healing and it would be interesting to study cell interactions (64) and the effect on wound parameters.

In conclusion, our developed FSE incorporating monocytes and T cells represents a significant step towards the development of a more realistic skin model that allows the study of innate and adaptive immune reactions related to burn injury, while avoiding the use of experimental animals. Ultimately, our immunocompetent model has the potential to advance the study of therapeutics modulating inflammatory reactions in burned skin to improve wound healing.

## Data availability statement

The raw data supporting the conclusions of this article will be made available by the authors, without undue reservation.

## Ethics statement

Ethical approval was not required for the studies involving humans because in this study, tissue samples were obtained from planned elective plastic surgeries (healthy skin) that were part of routine patient treatment. Therefore, these tissues are residual materials and no evaluation by the medical ethical committee was required for the collection of these samples. As described in the Materials and Methods section, we used an opt-out protocol to receive tissue samples. Subjects were informed of this procedure and were able to withdraw at any point. This procedure is in accordance with the national guidelines (https://www.coreon.org/) and institutional guidelines of the local hospitals in Beverwijk. Buffy coats from healthy donors were purchased at the Dutch Blood bank (Sanquin, Amsterdam, the Netherlands), in accordance with the national and institutional guidelines. The studies were conducted in accordance with the local legislation and institutional requirements. Written informed consent for participation was not required from the participants or the participants’ legal guardians/next of kin in accordance with the national legislation and institutional requirements because in this study, tissue samples were obtained from planned elective plastic surgeries (healthy skin) that were part of routine patient treatment. Therefore, these tissues are residual materials and no evaluation by the medical ethical committee was required for the collection of these samples. As described in the Materials and Methods section, we used an opt-out protocol to receive tissue samples. Subjects were informed of this procedure and were able to withdraw at any point. This procedure is in accordance with the national guidelines (https://www.coreon.org/) and institutional guidelines of the local hospitals in Beverwijk. Buffy coats from healthy donors were purchased at the Dutch Blood bank (Sanquin, Amsterdam, the Netherlands), in accordance with the national and institutional guidelines.

## Author contributions

PM: Conceptualization, Data curation, Formal Analysis, Funding acquisition, Investigation, Methodology, Project administration, Software, Supervision, Visualization, Writing – original draft. MV: Conceptualization, Formal Analysis, Investigation, Methodology, Software, Validation, Visualization, Writing – review & editing. AE: Formal Analysis, Investigation, Methodology, Validation, Writing – review & editing. LR: Formal Analysis, Investigation, Software, Visualization, Writing – review & editing. LM: Formal Analysis, Investigation, Visualization, Writing – review & editing. EM: Conceptualization, Formal Analysis, Funding acquisition, Methodology, Project administration, Resources, Supervision, Writing – review & editing. IJ: Conceptualization, Formal Analysis, Funding acquisition, Methodology, Supervision, Writing – review & editing. HK: Conceptualization, Formal Analysis, Funding acquisition, Methodology, Supervision, Writing – review & editing. BB: Conceptualization, Formal Analysis, Funding acquisition, Methodology, Project administration, Resources, Supervision, Writing – review & editing.

## Supplementary material

Supplementary Figure 1 Gating strategy. Gating strategy is shown for **(A)** monocyte-derived macrophage panel and **(B)** T cell panel.

Supplementary Figure 2 Cytokine levels (IL-2, IL-17A, MCP-1, TNF-α) in medium of (burn-injured) FSEs after 7 days of culture with monocytes. Samples from biological duplicates were averaged per donor. Concentrations are reported in pg/mL medium. Experiments were performed in duplicate using 6 different keratinocyte donors, 6 fibroblast donors and 4 monocytes donors. The dashed line indicates the lowest level of quantification and the highest limit of quantification (for MCP-1). Statistically significant differences were calculated using Mann-Whitney U test. Only comparisons between uninjured and burn-injured models or between models without and with monocytes are shown. Significant differences are indicated by asterisks: *: p < 0.05.

Supplementary Figure 3 Cytokine levels (IL-1β, IL-2, MCP-1, TNF-α) in medium of (burn-injured) FSEs after 7 days of culture with pre-activated T cells. Samples from biological duplicates were averaged per donor. Concentrations are reported in pg/mL medium. Experiments were performed in duplicate using 6 different keratinocyte donors, 6 fibroblast donors and 5 T cell donors. The dashed line indicates the lowest level of quantification and the highest limit of quantification (for MCP-1). Statistically significant differences were calculated using Mann-Whitney U test. Only comparisons between uninjured and burn-injured models or models without and with T cells are shown. Significant differences are indicated by asterisks: *: p < 0.05.
