# Supplementary figures and images for: Monocytes and T cells incorporated in full skin equivalents to study innate or adaptive immune reactions after burn injury
Source: Front Immunol. 2023 Oct 13;14:1264716. doi: 10.3389/fimmu.2023.1264716 (PMC10611519; doi:10.3389/fimmu.2023.1264716)

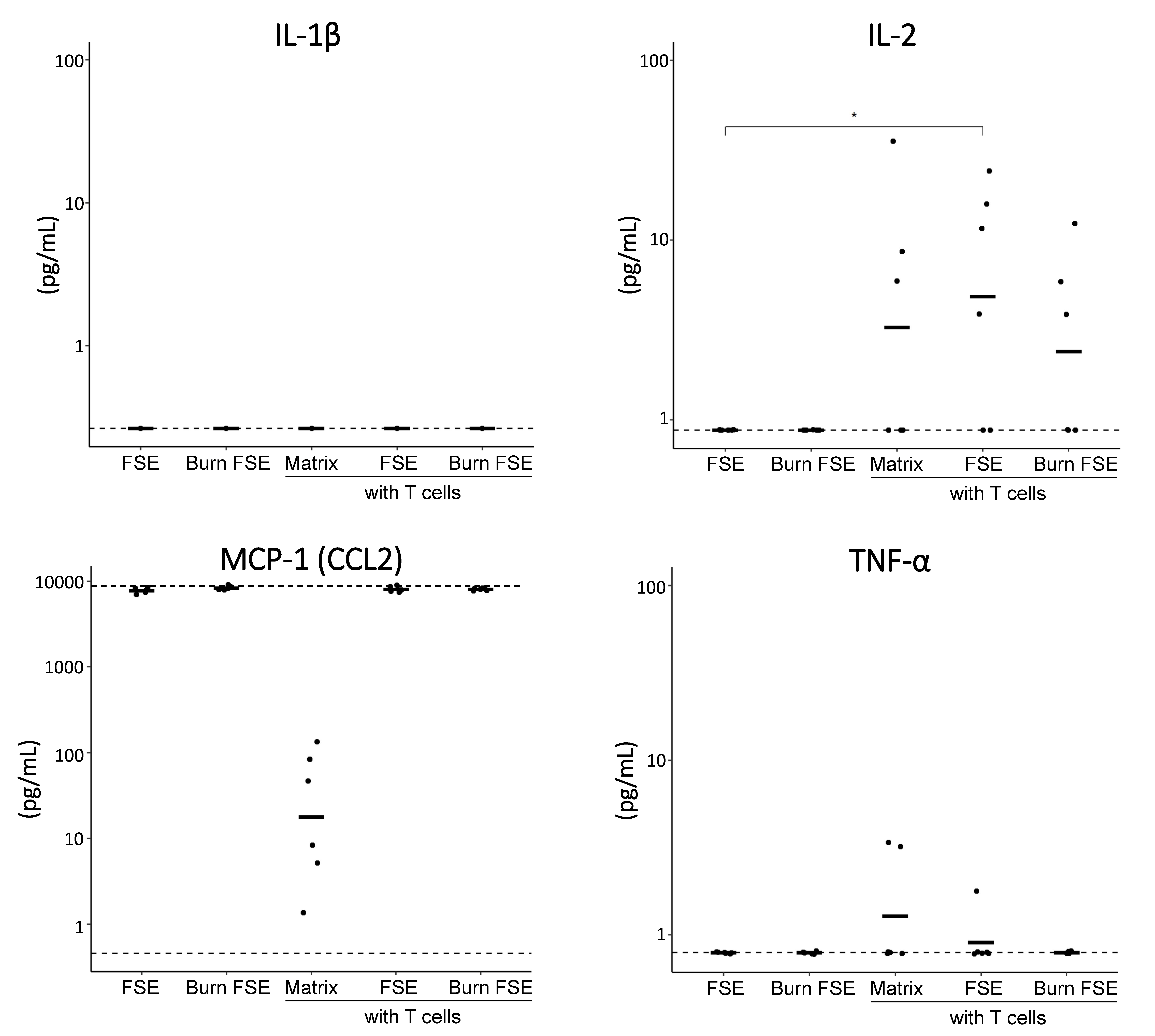

Supplement: Supplementary Figure 3 — Cytokine levels (IL-1β, IL-2, MCP-1, TNF-α) in medium of (burn-injured) FSEs after 7 days of culture with pre-activated T cells. Samples from biological duplicates were averaged per donor. Concentrations are reported in pg/mL medium. Experiments were performed in duplicate using 6 different keratinocyte donors, 6 fibroblast donors and 5 T cell donors. The dashed line indicates the lowest level of quantification and the highest limit of quantification (for MCP-1). Statistically significant differences were calculated using Mann-Whitney U test. Only comparisons between uninjured and burn-injured models or models without and with T cells are shown. Significant differences are indicated by asterisks: *: p < 0.05. [file Image_3.tif]
