# Supplementary material for: Monocytes and T cells incorporated in full skin equivalents to study innate or adaptive immune reactions after burn injury
Source: Front Immunol. 2023 Oct 13;14:1264716. doi: 10.3389/fimmu.2023.1264716 (PMC10611519; doi:10.3389/fimmu.2023.1264716)
Supplement: Supplementary file 4 [file Table_1.docx]

**SUPPLEMENTARY TABLES**

**Supplementary Table 1. Culture media used for cell and FSE culture.**

| Medium | Contents |
| --- | --- |
| Culture medium | Dulbecco’s Modified Eagle’s Medium (DMEM), 10% fetal calf serum (Fetalclone III, Logan, UT); 1% 200 mM glutamine, antibiotics (100 IU/mL penicillin, 100 µg/mL streptomycin (all Invitrogen)) |
| FSE I medium | DMEM + Ham’s F12 Nutmix (3:1) (Invitrogen, Paisley, UK), 5% fetal calf serum (Fetalclone III), 1.1 µM hydrocortisone, 1 µM isoproterenol, 0.09 µM insulin, a lipid supplement (25 µM palmitic acid, 15 µM linoleic acid, 7 µM arachidonic acid, and 24 µM bovine serum albumin (all Sigma-Aldrich)), antibiotics (100 IU/mL penicillin, 100 µg/mL streptomycin) |
| FSE II medium | DMEM + Ham’s F12 Nutmix (3:1) (Invitrogen), 2% fetal calf serum (Fetalclone III), 1.1 µM hydrocortisone, 1 µM isoproterenol, 0.09 µM insulin, 1.9 μM DL-α-tocoferol, 5.01 µM β-cyclodextrin, 10.1 µM L-carnitine, 9.99 µM serine, a lipid supplement (25 µM palmitic acid, 15 µM linoleic acid, 7 µM arachidonic acid, and 24 µM bovine serum albumin), antibiotics (100 IU/mL penicillin, 100 µg/mL streptomycin) |
| FSE III medium | DMEM + Ham’s F12 Nutmix (3:1) (Invitrogen), 0.5% fetal calf serum (Fetalclone III), 1.1 µM hydrocortisone, 1 µM isoproterenol, 0.09 µM insulin, 1.9 μM DL-α-tocoferol, 5.01 µM β-cyclodextrin, 130 µg/mL ascorbic acid, 10.1 µM L-carnitine, 9.99 µM serine, a lipid supplement (25 µM palmitic acid, 15 µM linoleic acid, 7 µM arachidonic acid, and 24 µM bovine serum albumin (all Sigma-Aldrich)), antibiotics (100 IU/mL penicillin, 100 µg/mL streptomycin) |

**Supplementary Table 2. Antibodies used for flow cytometry.**

| Panel | Primary antibody | Clone | Conjugate |  |
| --- | --- | --- | --- | --- |
| Macrophage | anti-CD11b | REA713 | FITC | Miltenyi Biotec GmbH |
|  | anti-CD14 | REA599 | VioBlue |  |
|  | anti-CD16 | REA423 | APC |  |
|  | anti-CD68 | REA886 | APC-Vio 770 |  |
|  | anti-CD163 | REA812 | PE |  |
|  | anti-HLA-DR | L243 | PerCP/Cyanine5.5 | BioLegend |
| T cell | anti-CD3 | REA613 | APC-Vio770 | Miltenyi Biotec GmbH |
|  | anti-CD4 | REA623 | VioBlue |  |
|  | anti-CD25 | REA945 | PE-Vio770 |  |
|  | anti-CD127 | REA614 | VioBright FITC |  |
|  | anti-CD183 (CXCR3) | G025H7 | Briljant Violet 510 | BioLegend |
|  | anti-CD194 (CCR4) | REA279 | PE | Miltenyi Biotec GmbH |
|  | anti-CD196 (CCR6) | REA190 | APC |  |

**Supplementary Table 3. Antibodies used for immunohistochemistry.**

| Primary antibody | Clone | Host | Dilution | Manufacturer | Antigen retrieval |
| --- | --- | --- | --- | --- | --- |
| anti-CD3 | Sp7 | Rabbit | 1/200 | Abcam | 10 min in EDTA (Fisher) of pH 9 at 70 °C |
| anti-CD14 | Sp192 |  | 1/150 | Sigma-Aldrich | 20 min in Sodium Citrate (Fisher) of pH 6 at 70 °C |
| anti-CD68 | KP1 | Mouse | 1/2000 | DAKO | 10 min in EDTA of pH 9 at 70 °C |
| BrdU | IIB5 |  | 1/200 | MP Biomedicals | 30 min in HCl (Fisher) and 2 times 5 min in Borax (Fisher) of pH 8.5 at RT |
